# The Impact of Thyroid Diseases on the Working Life of Patients: A Systematic Review

**DOI:** 10.3390/ijerph17124295

**Published:** 2020-06-16

**Authors:** Veruscka Leso, Ilaria Vetrani, Luigi De Cicco, Alessandro Cardelia, Luca Fontana, Gaetano Buonocore, Ivo Iavicoli

**Affiliations:** 1 Section of Occupational Medicine, Department of Public Health, University of Naples Federico II, Via S. Pansini 5, 80131 Naples, Italy; veruscka.leso@unina.it (V.L.); ilariavetrani20@gmail.com (I.V.); luigi_mario@hotmail.it (L.D.C.); alecardiff@gmail.com (A.C.); ivo.iavicoli@unina.it (I.I.); 2 Clinical Directorate, University Hospital Federico II, Via S. Pansini 5, 80131 Naples, Italy; gaetano.buonocore@unina.it

**Keywords:** thyroid cancers, hyperthyroidism, hypothyroidism, occupation, work ability, professional life

## Abstract

Thyroid diseases are characterized by a wide range of physical and mental symptoms that can affect biological function, emotional and social life of patients. However, their impact on work functioning is not yet fully understood. Therefore, this review aims to address the way in which thyroid diseases can affect occupational outcomes, i.e., the employment rate, sick leave, working capacity and work income of patients. A systematic review of Pubmed, Scopus and ISI Web of Knowledge databases has been performed. Although it is not possible to extrapolate precise data for benign pathologies, about a third of the survivors of thyroid cancer could be unemployed. Hyperthyroid and hypothyroid patients presented a greater risk of long-term sick leave than controls, depending on the severity of the disease. Hyperthyroidism impaired working ability in about a third of affected patients, particularly in cases complicated by orbitopathy with diplopia. A possible influence of thyroid diseases on various occupational outcomes emerged from our review, however further research seems necessary to understand the relationship between work problems, specific pathological characteristics over time and risk factors in the workplace. This may support a comprehensive, interdisciplinary management of thyroid disorders, with benefits for patients’ personal, social and professional life.

## 1. Introduction

Thyroid diseases include benign functional, inflammatory, autoimmune and neoplastic diseases characterized by a wide range of physical and mental symptoms primarily, but not exclusively, related to the excess or to the deficiency of circulating thyroid hormones, indeed to the hyper- or hypo-thyroid conditions they can cause [1,2,3,4,5,6]. Such alterations may not only affect the biological and functional aspects of the organism, but may also have an impact on emotional, relational, social, and working life of affected individuals [7]. In this context, the type of thyroid disease, related symptoms and the medical-surgical treatments adopted may all play an influencing role on the patients’ quality of life. According to the health and disability model, the consequences of diseases are health-related barriers which prevent an individual from fully exploiting his/her daily activities, as well as from participating in various social roles, including work [8]. This model proposes that the impact of disease on work functioning, along with other individual and environmental factors, can lead to temporary or permanent exclusion from the labour market, due to unemployment, sickness absence, or disability pensioning. Additionally, the health status of patients can strongly influence their ability to work, intended as the dynamic concept addressing the compatibility between work demands and individual resources [9]. Although many patients become ill with thyroid pathologies during their working age, limited information is currently available concerning the quality of their overall working life [7]. Workers suffering from thyroid pathologies, may be forced to live with physical, psychic, psychosocial and psychosomatic problems, which may limit their work ability, causing absenteeism and consequently difficulty in maintaining their jobs [10,11]. Additionally, it remains to be explored the impact that also adequately treated pathologies may have on work. This seems an even more important aspect to be understood, taking into-account that maintaining a good working capacity, in relation to health and employment conditions, translates into a better quality of life, thus improving the comprehensive management of the thyroid disorders [12,13]. Therefore, the aim of our review was to address the impact that different thyroid diseases may have on patients’ work functioning, in terms of employment rate, risk of sick leave, patients’ work ability, as well as disease consequences on labour market income. In the perspective to achieve a balance between job productivity and work sustainability for affected patients, it appears relevant to understand how thyroid pathologies may influence occupational outcomes. In this regard, specific attention should be focused at defining which pathological features, including also extra-thyroidal complications and therapeutic options may differently impact the working lives of patients and which occupational risk factors may be experienced as more difficult to face according to the various pathological conditions. Overall, this may support, a more adequate risk assessment process in the workplace, specifically focused at conditions of hyper-susceptibility and a more comprehensive, interdisciplinary management of the diseases with the aim to improve patients’ health outcomes, but also the quality of their personal, social and professional life.

## 2. Materials and Methods

The Preferred Reporting Items for Systematic Reviews and Meta-Analyses Statement (PRISMA) criteria were followed to perform a systematic literature search [14]. Studies addressing possible implications of “thyroid diseases on the professional life” of patients published until 9 April 2020 were identified by research on three principal scientific databases: PubMed, Scopus, and ISI Web of Science. The term “thyroid” was combined through the “AND” Boolean operator with “work” or “occupation” in order to assess the impact of thyroid diseases on work functioning. The two lines were additionally combined with the operator ”AND” with the keywords: “employment” or “work ability” or “disability” or “sick leave” or “income” employed to describe the outcomes of the work impact of the diseases. Three of the authors independently reviewed all the titles and abstracts retrieved through the computerized search and selected papers suitable for the review purposes according to the inclusion criteria. These regarded all types of human peer-reviewed research articles including descriptive epidemiological-occupational surveys, medical reports, case series, cohort and case-control studies published in English, and reporting possible implications of “thyroid diseases” on employment, sick leave, work ability and labour market income in patients. Exclusion criteria regarded reviews, case reports, conference papers, and publications that did not focus on work consequences of thyroid pathologies or that were published in languages other than English. Two of the authors independently assessed the quality of the studies included in the review. A methodological critical evaluation of each individual study was performed using the Newcastle Ottawa scale (NOS) adapted for cross-sectional studies for systematic review [15,16]. Adapted NOS assesses the risk of bias in three areas: selection of participants (maximum 5 points); study group comparability (maximum two points); and evaluation of results (maximum 3 points). The quality assessment of the studies was rated as follows: very good (9, 10 points); good (7, 8 points); satisfactory (5, 6 points), unsatisfactory (0–4 points) and included in Tables 1–4. 

## 3. Results

**Figure 1:**
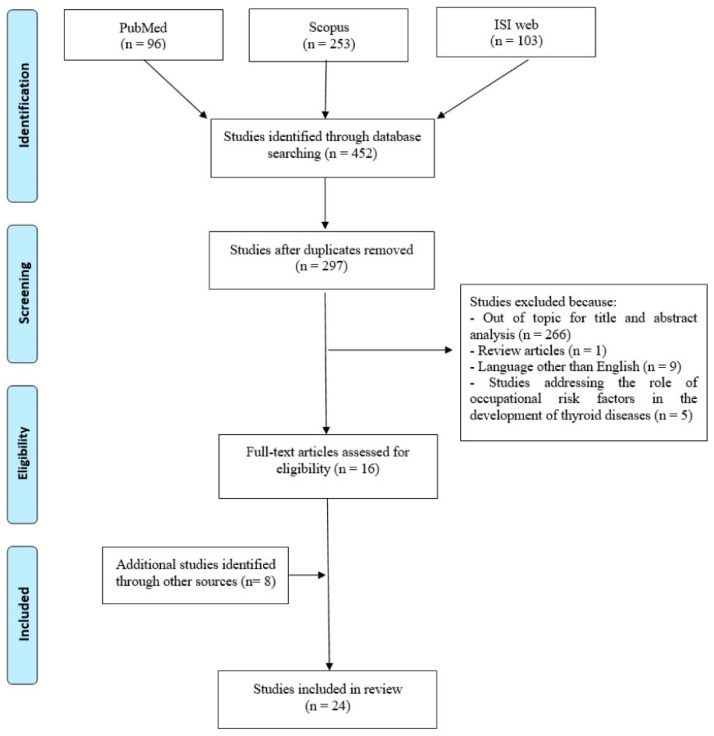
Flow diagram of literature search.

The preliminary search retrieved a total of 452 articles: 96, 253 and 103 references through PubMed, Scopus and ISI Web of Science databases, respectively. After removal of duplicates, 297 articles remained. Among those, studies that did not meet the inclusion criteria were excluded according to the following reasons: 266 were removed because considered out of the topic from the title or abstract analysis, 1 because it was a review article, 9 studies were in a language other than English. Five additional studies were excluded because they focused on the possible role of occupational risk factors on the development of thyroid diseases. After removal of these, 16 articles remained. By assessing the reference list accompanying the selected articles the citation pool of relevant publications was further enlarged through the identification of 8 additional eligible papers. Overall, our search retrieved a total of 24 publications for review (Figure 1). Retrieved studies could generally demonstrate the impact of thyroid disorders, primarily functional hyper- or hypo-thyroid alterations, with related extra-thyroidal manifestations, as well as of malignant pathologies, on different work-related outcomes: employment status, sick leave, work ability, and labour income of affected patients. Additionally, some disease features and therapeutic options have been reported to function as factors potentially influencing professional life issues of patients as detailed in the following paragraphs.

### 3.1. Thyroid Disease and Employment Status

The working status is a fundamental aspect in the life of thyroid patients that affects their quality of life [10,17]. In a longitudinal, health register-based study that followed-up thyroid patients affected by non-toxic goiter, hyperthyroidism, Graves’ orbitopathy (GO) and autoimmune hypothyroidism from 1994 to 2011, no differences in the risk of transition from work to unemployment could be detected compared to controls in the first year after diagnosis [10]. Comparably, in this same period, no significant differences were detected in the transition from unemployment to work comparing thyroid patients to controls. In the following years, a significant difference was reported due to the lower probability to return to work for patients with GO [10]. In such kind of patients, a gainful employment was demonstrated in 77% of cases by Ponto et al. [18], with a greater percentage of employed workers in the eu-hypothyroid group (95%), compared to the hyperthyroid one (74.6%) [11]. A lower percentage of employed workers (35%) was reported by Fahrenfort et al. [19] among a population of 303 patients treated for hyperthyroidism in the period 1994–2000 (Table 1).

Concerning differentiated, papillary and follicular thyroid cancers, the 5 and 10-year survival rates exceed 90% and 70%, respectively [20]. Therefore, for all patients who have developed cancer at working age and survived the disease, the ability to maintain their occupational role represents an important aspect. Many cancer survivors are forced to change jobs or work part-time [21], have problems in returning to work [22,23] and experience financial difficulties [24]. Dueren et al. [25] analyzed a group of differentiated thyroid cancer (DTC) patients treated with thyroidectomy and radioiodine ablation. Data concerning occupational activity showed that 55.2% of them were employed, 3.2% were jobless, while 1.6%, 15.2% and 24.8% were trainees or students, housewives and pensioners, respectively. These results are in line with those previously reported by Tan et al. [26], Schultz et al. [27] and Borget et al. [28] who found that 53.8%, 63% and 66% of the cancer patients investigated, respectively, had a remunerated activity. Tamminga et al. [29] examined the consequences of a diagnosis of a papillary, follicular or medullary thyroid cancer on work outcomes in 223 survivors followed between 1990 and 2008. Seventy-one per cent of the survivors were employed. In the unemployed subgroup, the reasons for not having a work activity or function were disability (33%), early retirement (e.g., due to reconstitution) (14%), no job (6%), or other (voluntary unemployed) (46%). A quite lower rate of employment was reported by Luster et al. [30] who reported that 46% of patients with differentiated thyroid carcinomas were salaried employees. An increased risk of unemployment in thyroid cancer survivors compared to controls was also reported in an Israelian study [31]. While a positive association was found between thyroid cancer and risk of unemployment 2 years after diagnosis, such relationship lost significance 4 years after diagnosis. Analysis of the data by gender showed a significantly higher risk of being unemployed only among women, and this finding was confirmed also when ethnic and education variables were analyzed. A more recent investigation reported that, among 126 survivors of thyroid cancer, 104 (82.5%) were employed sometime between the diagnosis and the study participation period [32]. Mongelli et al. [33] reported that about a half of the 1753 investigated cancer survivors were employed full time, 10.6% had a part time employment, while the 4.8% reported being self-employed or a small business owner. The unemployment rate was 12.2%. Additionally, in the study, 18.1% (315) of patients reported past unemployment for 6 months or more due to thyroid cancer or related treatment. No significant differences between thyroid carcinoma patients and their partners was reported by Neudeck et al. [34] (Table 1).

Factors associated with unemployment in cancer survivors were: a higher age at the time of the survey, a lower education level, an unfavorable cancer stage, a higher level of fatigue, anxiety and depression, and comorbidities [29]. These findings are in line with those by Ratzon et al. [31] that previously reported age as a significant risk factor for unemployment, while education and a high socioeconomic status as protective factors. Additionally, being unemployed 2 years before diagnosis was significantly associated with unemployment at 2 and 4 years afterwards. Concerning the predictive role of fatigue for unemployment suggested by Tamminga et al. [29], a substantial percentage of thyroid cancer survivors were reported to claim with moderate or severe fatigue by Ahmad Alhashemi et al. [35]. Unemployed subjects had significantly more tiredness compared to employed persons, housewives/caregivers, students, and pensioners. However, a definite causal relationship between these two aspects could not be extrapolated. As a further confirmation, Tan et al. [26] showed that thyroid cancer survivors who have been working showed a better physical and emotional role, suggesting that patients able to continue to work were those less affected by the disease, and also that work may have beneficial effects on the physical and emotional aspects of patients’ life.

**Table 1:** Studies addressing the impact of thyroid disease on employment status.

Study Location (Analysed Period)	Population Investigated (Number) and Age	Additional Information	Results	Quality Rating According to NOS	References
Copenaghen, Denmark (2007–2013)	Thyroid patients (n. 632; F: n. 88; age between 18 and 59 years) Controls: n. 15,050 from the general Danish population Two different time points for the analysis: 2007–2008 (time 1); 2012–2013 (time 2).	Clinical data Patients with diagnosis of: non-toxic goiter, toxic nodular goiter, Graves’ disease (with or without orbitopathy), autoimmune hypothyroidism, and other thyroid diseases (for example, postpartum thyroiditis and subacute thyroiditis).	Employment status: employed thyroid patients (n. 507). Participants who reported thyroid associated work limitations at time 1 were 5 times more likely to be excluded from the labor market at time 2 (OR 5.0, 95% CI 2.7–9.1).	Satisfactory	Nexo et al. [10]
Mainz, Germany (2005–2012)	TAO patients (n. 461; F: n. 360).	Clinical data Hormonal status: hypothyroid (n.12; F: n.12; median age: 51 years); euthyroid: (n. 8; F: n. 7; median age: 55 years); hyperthyroid (n. 441; F: n. 341; median age: 49 years)	Employment status: of patients with eu-/hypothyroid TAO 19 of 20 (95%) and 329 of 441 hyperthyroid (74.6%), respectively, were working.	Good	Ponto et al. [11]
Mainz, Germany (2006–2008)	Outpatients with EO (n. 250; M: n. 41; F: n. 209; Median age: 49 years, range: 13–81 years)	Clinical data EO severity assessed according to: retraction of the eyelid, eyelid beat rate, inflammation of affected soft tissues, proptosis, diplopia, and affection of the cornea and the optic nerve *Occupational data* Questionnaire survey including items on unfit to work, earning capacity and psychotherapy	Gainful employment n. 192 (77%); lost job n. 5 (2.6%), retired early n. 10 (5.2%)	Satisfactory	Ponto et al. [18]
Amsterdam, Netherlands (1994–2000)	Patients treated for hyperthyroidism (n. 303; F: 87%; mean age: 44.0 ± 11.2 years).	Clinical data Hyperthyroidism treatment: surgery (n. 31; 10%); 131I (n. 151; 50%); thyro-static drugs (n. 78; 26%); levothyroxine (n. 166; 55%). A total of 119 patients (40%) reported no drug treatment at the survey time.	Employment status: full-time job (n. 107; 35%).	Satisfactory	Fahrenfort et al. [19]
Wuerzburg, Germany (2008)	Differentiated thyroid carcinoma patients (n. 128; F: 75.8%; M: 24.2%; Mean age: 51.6 ± 14.8 years)	Clinical data Treatment: thyroidectomy followed by radioiodine. Control visits including I-131 WBS: the first control (after 3–6 months) included a hypothyroidism phase with 2–5 week of suspension of hormone therapy; the second (6–12 months after the first) included exogenous administration of rhTSH in euthyroidism. Occupational data Available for n. 125 patients.	Employment status: employed 55.2%; trainees or students 1.6%; unemployed 3.2%; housewives 15.2%; retired 24.8%.	Satisfactory	Dueren et al. [25]
Singapore (2000)	Patients with differentiated thyroid cancer (n. 144; F: n.107, mean age: 47.8 ± 13.7 years)	Clinical data Most of patients with differentiated cancers had total thyroidectomy and were subsequently referred for radioiodine therapy.	N. 77 (53.8%) worked full time or part time; n. 67 (46.2%) were unemployed or retired. Employed survivors scored better at items “role physical and emotional” at the quality of life assessment.	Unsatisfactory	Tan et al. [26]
Houston, Texas	Thyroid cancer survivors (n. 518; F: n. 426; M: n. 92; mean age: 37.9 ± 11.6 years)	Clinical data The mean ± SD age at diagnosis was 37.9 ± 11.6 years. All participants had been treated with surgery and 417 (80.5%) had been treated also with radiation.	Among n. 518 survivors, n. 324 (63%) were employed.	Good	Schultz et al. [27]
Villejuif, Saint-Cloud, Lille, France (2004–2006)	Thyroid carcinoma patients (papillary or follicular) (n. 292; F: 74%; mean age: 46.7 ± 13.2 years). Pre WBS-stimulation by withdrawal (n. 119; F: 78%; mean age 47.7 ± 13.6 years); rhTSH (n. 173; F: 71%; mean age: 46 ± 12.8)	Clinical data Patients treated with thyroidectomy and radioiodine ablation. Diagnostic WBS was performed in all patients who had undergone withdrawal and in 42% of patients who had undergone rhTSH stimulation.	N. 194 (66%) active patients: n. 176 patients with a remunerated activity and n. 18 job seekers. The other n. 98 patients were retired (n. 50), had no professional activity (n. 36), were students (n. 7) or disabled (n. 3).	Good	Borget et al. [28]
Amsterdam, Gronigren, Nijmegen, Eindhoven, The Netherlands (1990–2008)	Thyroid cancer survivors (n. 223; M: n. 49; mean age: 49.5 ± 9.8 years)	Clinical data Primary treatment surgery (29%); surgery followed by 131 I therapy: (70 %). Median time since diagnosis: 9.0 years.	Employment status: employed (71%). Reason for no employment: no job (6%), disabled (33%), early retirement (14%), other and probably volunteers (46%) Clinical factors associated with unemployment: higher age at time of survey, lower educational level, unfavourable cancer stage, higher level of fatigue, higher level of anxiety and of depression, comorbidities.	Good	Tamminga et al. [29]
Würzburg, Cologne, Germany (1992–2001)	Patients with differentiated thyroid cancers (n. 130; F: n. 88; median age: 52 years range 22–83)	Clinical data Patients underwent primary surgery and radioiodine ablation. Preparation for full WBS included thyroid hormone withdrawal of levothyroxine for at least 4 consecutive weeks and of triiodothyronine for at least the last 2 weeks.	N. 60 (46%) patients were salaried employees (outside home)	Satisfactory	Luster et al. [30]
Israel (1998–2011)	Thyroid cancer patients (n. 417; F: n. 330; mean Age: 43.5 ± 10.7 years) Control group (n. 1277; F: n. 1017; mean age: 43.8 ± 10.6 years)	Occupational data About a third of the participants in both groups did not work 2 years before diagnosis. The reduction in income was assessed between cases and controls after excluding those unemployed at the baseline (n. 1231).	2 years after diagnosis, the percentage of unemployed was significantly higher among cases than controls (39% vs. 29.2%, respectively). No significant differences were detected 4 years after diagnosis (33.6% vs. 29.9%, respectively).	Very good	Ratzon et al. [31]
United States (2019)	Thyroid cancer survivors (n. 126)	Clinical data Diagnosis of malignancy was made between 8 and 39 years. Survey completion was performed within 1 to 5 years from diagnosis and ≥ 1 year after therapy completion.	N. 104 (82.5%) were employed between cancer diagnosis and the survey.	Good	Ketterl et al. [32]
Chicago, United States	Thyroid cancer survivors (n. 1.743; F: 88%; mean age 51 ± 13 years)	Clinical data Papillary thyroid cancer (85%), follicular thyroid cancer (6.3%), medullary cancer (4.8%), non-invasive follicular thyroid neoplasm with papillary-like nuclear features (0.8%), reported anaplastic thyroid cancer (0.5%).	Employment status: full time employed n. 789 (45.3%); part-time employed n. 184 (10.6%); self-employed or small business owners n. 84 (4.8%). Unemployed n. 213 (12.2%); retired n. 323 (18.5%), students n. 31 (1.8%). Lost employment during course of thyroid cancer diagnosis and treatment n. 232 (13.3%).	Satisfactory	Mongelli et al. [33]
Olten, Zurich, Luzern, Switzerland (2009–2016)	Thyroid carcinomas patients (n. 66; F: n. 46, M: n. 20; mean age 45.77 ± 10.4 years). Controls (n. 38; F: n. 12; M: n. 26; mean age: 46.14 ± 11.0 years)	Clinical data Patients diagnosed and treated (by operation and radioactive iodine treatment) over the previous 7 years. Occupational data Questionnaire survey including data on days to return to work after hospital discharge; work-related impairment and reasons; current work ability and workload; sick leave.	No significant difference between patients (54 of 66; 81.8%) and partners (32 of 38; 84.2%).	Satisfactory	Neudeck et al. [34]
Toronto, Canada (2015)	Thyroid cancer patients (n. 205; F: n. 152; Median age 52.5 ± 13.5 years)	Clinical data Differentiated thyroid cancer: n. 195. 83 (89.3%) patients treated with total thyroidectomy (completed in one or two stages) with or without neck nodal dissection.	Employment status: unemployed or unable to work due to disability (11.8%); employed (61.3%); fulltime homemaker/caregiver (4.4%); retired: (19.6%). Clinical factors significantly associated with unemployment, impaired work ability: worsening fatigue.	Satisfactory	Alhashemi et al. [35]

EO, endocrine orbitopathy; GO, Graves’ orbitopathy; OA, old adults; RhTSH, recombinant human thyroid stimulating hormone; TAO, thyroid-associated orbitopathy; WBS, whole body scan; YA, young adults.

### 3.2. Thyroid Diseases and Sick Leave 

Days lost from work are a common proxy for morbidity (Table 2). Among the thyroid pathologies that may determine a serious hormonal imbalance, hyperthyroidism, a condition characterized by an excessive production of thyroid hormones, may cause symptoms, such as anxiety, irritability and nervousness that may impact patients’ work schedules [10,11,36]. Nexo et al. [10] found a significantly higher risk of long sick leave in patients with hyperthyroidism within the first year after the diagnosis compared to controls, together with a significantly lower probability to return to work in the subsequent years. A Swedish study compared the risks and benefits of three types of treatment of Graves’ hyperthyroidism: (i) antithyroid drugs, (ii) medical or subtotal thyroidectomy, (iii) medical surgical or radioiodine-131 treatment by assessing the impact on absenteeism at work [37]. The authors found no significant differences between the differently treated groups in the number of days patients stayed home from work due to the disease, analyzing the first 18 months after the start of treatment in comparison to the following 6 months [37]. This may suggest that sick leave may better reflect the severity of the disease and its impact on the patient health, while no differences seemed related to the treatment mode. 

One of the conditions that has been evaluated for its possible impact on the working life of patients is GO, the most common extra-thyroidal manifestation of autoimmune Graves’ hyperthyroidism characterized by disfiguring proptosis and diplopia [38]. Nexo et al. [10] found a significantly higher risk of long-term absence in GO patients within the first year of diagnosis, and the lower probability of returning to work in subsequent years. In a German study [36], the mean duration of sick leave in GO patients was 22.3 d/year compared to the average of 11.6 d/year of the general German population. Fifteen percent of patients had also taken longer sick leaves whose duration correlated with the GO severity. In fact, 40%, 17% and 8% of patients with sight-threatening, moderate to severe and mild vision-threatening GO, respectively, had taken more than 11.6 d/year of sick leave. In line with the idea that the disease severity may affect patients’ absenteeism, a 15% significantly lower risk of sick leave was reported in GO patients in a state of eu/hypothyroidism than in those in hyperthyroidism [11]. Hypothyroidism is a clinical condition that can cause somatic and psychiatric symptoms able to interfere with the normal performance of patients’ daily and working activities [10]. Additionally, hypothyroidism may have a major impact on work schedules among thyroid cancer survivors [39,40]. Patients with autoimmune hypothyroidism showed a significantly diminished probability to return to work after sickness absence in the first year after diagnosis [10]. The quantitative effects of hypothyroidism secondary to thyroid hormone withdrawal in patients with DTC was investigated by Luster et al. [30]. In this study, the authors found that hypothyroid patients experienced an average of 11 days of absence from work during the weeks of hormone withdrawal. Among 60 employed patients, 62% and 30% reported to have lost at least 4, or ≥30 working days, respectively, during the last phase of hypothyroidism [30]. However, such investigation did not focus on the risk of sick leave for patients receiving the recombinant human thyroid stimulating hormone (rhTSH) therapy as a stimulation procedure to thyroid hormone withdrawal in the diagnostic follow-up of thyroid cancer patients. To overcome this gap, Borget et al. [28] demonstrated that patients treated with rhTSH, avoiding periods of hypothyroidism, were less likely to require sick leave, and showed a lower duration of absence, compared to patients suffering hormone withdrawal for 3–5 weeks. Specifically, from 30 days before to 30 days after control, a significantly higher percentage of patients treated by rhTSH did not need sick leave during the entire period compared to withdrawal stimulated patients (89 vs. 67%, respectively). The rhTSH stimulation allowed a reduction of 8.1 days of sick leave length per active patient [28]. Dueren et al. [25] analyzed a group of DTC patients treated with thyroidectomy and radioiodine ablation and followed up at 3–6 months post-thyroidectomy after a phase of thyroid hormone withdrawal and again after 6–12 months later in a euthyroid state under exogenous stimulation with rhTSH therapy. In the four weeks before the thyroid hormone withdrawal period, 47.8% of investigated patients had been absent from their work for a median of 10 days, while following this period, 37.5% needed sickness absence for a median of 6 days. Lower percentages of absence were evident before and after the period of rhTSH treatment with 4.5% (mean absence of 4 days) and 7.7% (median of 5 days of absence) percentages of absent patients, respectively. Among thyroid cancer survivors in a US study, 73.7% reported to have taken time away from work due to their thyroid cancer diagnosis or related treatment [33]. Neudeck et al. [34] reported that male patients, after hospital discharge following cancer operation and radioactive iodine treatment, returned to work after a median of 2 weeks, while female patients returned to work after a median of 3 weeks. In this regard, concerning the impact of different therapeutic cancer protocols in affecting work absenteeism, the mean duration of sick leave was significantly lower in patients with differentiated thyroid cancer who received rhTSH aided radioiodine ablative therapy (RIT) one week after thyroidectomy than in patients who received rhTSH aided RIT after 4–6 weeks following standard protocol [41]. Moreover, among the employed cancer survivors investigated by Ketterl et al. [32], those who had been exposed to radiation were significantly more likely to need a long period of paid work rest compared to those who had not been exposed to radiation. In regards to possible effects of complications from common thyroid surgical procedures on sickness absence, patients experiencing hypoparathyroidism, vocal cord paralysis, as well as vocal cord paralysis and concomitant hypoparathyroidism reported 43, 115 and 97 days lost from work [42].

**Table 2:** Summary of the studies addressing the impact of thyroid disease on sick leave.

Study Location (Analysed Period)	Population Investigated (Number) and Age	Additional Information	Results	Quality Rating According to NOS	References
Copenhagen, Denmark (1994–2011)	Hyperthyroid patients (n. 862; M: n. 97; F: n. 765) Controls (n. 7043; M: n. 833; F: n. 6210) Age range 20–59 years	Clinical data Patients had a diagnosis of: nontoxic goiter; hyperthyroidism by either nodular toxic goiter; GO; autoimmune hypothyroidism; other thyroid diseases (i.e., postpartum thyroiditis, subacute thyroiditis, or others)	Significantly increased risk in hyperthyroid patients (<1 year from the diagnosis) (HR 1.96; 95%CI 1.31–2.94) and GO patients’ (<1 year: HR 6.94; 95%CI 4.19–11.50; >1year HR 2.08; 95%CI 1.48–2.93).	Very good	Nexo et al. [10]
Stockholm, Uppsala, Sweden (1983–1990).	Graves’ hyperthyroid patients (n. 179; 60 patients between 20–34 years: young adults (YA); 119 patients between 35–55 years: old adults OA))	Clinical data YA received anti-thyroid drugs for 18 months (medical treatment) or subtotal thyroidectomy (surgical treatment). OA received medical, surgical, or radioiodine treatment. Follow-up time: at least 48 months.	No significant differences between the five treatment groups. Mean days of absence for the first 18 months after initiation of treatment vs. the following 6 months were: medical YA: 71 ± 21 vs. 12 ± 4; surgical YA: 65 ± 15 vs. 10 ± 3; medical OA: 62 ± 10 vs. 10 ± 4; surgical OA: 75 ± 12 vs. 10 ± 3; radioiodine OA: 74 ± 12 vs. 9 ± 3.	Satisfactory	Torring et al. [37]
Mainz, Hannover, Germany (2005–2009)	Patients with GO (n. 310; F: n. 259; M: n. 51; mean age: 48.6±13.7 years)	Clinical data The exclusion criteria were a missing signature and an uncertain diagnosis of GO.	Mean sick leave duration in GO patients: 22.3 ± 60.8 d/yr.	Satisfactory	Ponto et al. [36]
Mainz, Germany (2005–2012)	See Table 1	See Table 1	The risk of sick leave and work disability was 15% and 13% significantly lower in eu-/hypothyroid vs. hyperthyroid TAO patients.	Good	Ponto et al. [11]
Würzburg, Cologne, Germany (1992–2001)	See Table 1	See Table 1	Among employed patient, n. 37 (62%) reported missing at least 4 workdays during the last period of hypothyroidism with a median of 11 days; n. 11 (18%) reported missing ≥30 days of work.	Satisfactory	Luster et al. [30]
Villejuif, Saint-Cloud, Lille, France (2004–2006)	See Table 1	See Table 1	Duration of sick leave (days) was significantly lower in patients treated by rhTSH vs. patient treated by withdrawal (before control 1.4 ± 6.0 vs. 5.0 ± 10.2 days respectively; after control 1.7 ± 6.4 vs. 6.2 ± 10.7 days, respectively).	Good	Borget et al. [28]
Wuerzburg, Germany (2008)	See Table 1	See Table 1	4 weeks before control 1 47.8%; median of 10 days of absence (range 1–30 d). After control 1: 37.5% median of 6 days. Before control 2: 4.5% median 4 days (range 2–5); after control 2: 7.7% median 5 days.	Satisfactory	Dueren et al. [25]
Chicago, United States	See Table 1	See Table 1	Among thyroid cancer survivors, 73.7% reported to have taken time away from work due to their thyroid cancer diagnosis or related treatment	Satisfactory	Mongelli et al. [33]
Olten, Zurich, Luzern, Switzerland (2009–2016)	See Table 1	See Table 1	Patients returned to work after a median of 14 days; most partners continued to work immediately after the spouse’s diagnosis and treatment of DTC. Males returned to work after a mean of 2 weeks (mean 12.2) days and female after a mean of 3 weeks (mean 48.5 days).	Satisfactory	Neudeck et al. [34]
Utrecht, Netherlands (2013–2016)	Differentiated thyroid cancer patients (n. 20)	Clinical data Patients were treated with total or completion thyroidectomy followed by radioiodine therapy. Standard protocol (n. 9): rhTSH-aided radioiodine ablative therapy 4–6 weeks after surgery. Fast-track protocol (n. 11): rhTSH-aided radioiodine ablative therapy one week after thyroidectomy	Mean duration of sick leave was significantly lower in fast track group than in standard group (114.7 ± 58 vs. 280 ± 136 h, respectively)	Unsatisfactory	Waissi et al. [41]
United States (2019)	See Table 1	See Table 1	Radio-exposed subjects were significantly more likely to report taking a long period of paid work rest compared to those who not radio-exposed. No association between direct therapeutic exposures to cancer and interference with paid or unpaid work time.	Good	Ketterl et al. [32]
Kielce, Poland (2002–2007)	Patients affected by benign thyroid diseases treated with surgery (n. 756; F: n. 658; M: n. 98)	Clinical data Complications in n. 69 patients (9.1% of the total sample): hypoparathyroidism (n. 41, 5.4%), unilateral vocal cord paralysis (n. 17, 2.2%), bilateral vocal cord paralysis (n. 16, 2.1%).	Sick leave (among patients with complications): 74% continued to work after an average of 82 days of absence. Average absence duration after hospital discharge: vocal cord paralysis (114.7 days); concurrent vocal cord paralysis and hypoparathyroidism (97.5 days); hypoparathyroidism (43.1 days).	Unsatisfactory	Slaweta et al. [42]

GO, Graves’ orbitopathy; RhTSH, recombinant human thyroid stimulating hormone; TAO, thyroid- associated orbitopathy; YA, young adults.

### 3.3. Thyroid Diseases and Work Ability

Different studies addressed the impact of thyroid disease on patients’ ability to perform their job (Table 3). A Swedish study explored the work ability of 174 patients with Graves’ hyperthyroidism in the period between the disease manifestation to the beginning of treatment, as well as after the initiation of therapies [43]. Sixty-five percent of patients reported they could manage their professional work before the beginning of treatment, 63% indicated that therapies did not affect their working ability at all or very little, although 19% could not work, or work with severe limitations, for 1–3 months. In patients treated for Graves’ hyperthyroidism, over one third (35.3%) of those with a full-time job at the time of disease was unable to resume the same work even after remission of the disease [19]. Within this group, the 29.5% has been officially registered as completely or partially disabled. Interestingly, this study could point out the persistence of patients’ illness complaints, in terms of vegetative, cognitive neuro-psychological and emotional disturbances, although the remission of hyperthyroidism was achieved for at least 12 months. These included impaired memory, inaccuracy or forgetfulness worries, feeling hampered in carrying out variable tasks. Although a clear association was not investigated by the authors, a possible relationship between perceived complaints and occupational outcomes cannot be excluded. When the work ability of thyroid disease patients, including Graves’ disease, autoimmune hypothyroidism and other thyroid diseases not including goiters and GO, was assessed, affected individuals had significantly lower scores compared to the general population on the global work ability items. Within the first year of diagnosis, patients with Graves’ disease also rated their work ability to worsen with respect to mental demands [10]. Concerning the impact of extrathyroidal manifestations of Graves’ hyperthyroidism on work ability, a German study explored the occupational disability of 192 GO patients who were in gainful employment [18]. While, the majority (64.5%) were never considered unfit to work/disabled, more than one third of patients become unfit to work due to GO. Among those, 12 (6.2%) were permanently disabled, while 54 (29%) were temporary unfit to work (1 to 12 months). As predictive factors, the GO severity and diplopia resulted significantly associated with occupational disability, while no significant relationship was observed with proptosis. Importantly, about half of patients reported impairments to everyday functioning and/or to their own self-perception as a result of GO, and one fifth of GO patients had psychotherapy, particularly those who were unfit to work. Comparable percentages of disability were reported in a subsequent study of the same group [36] analyzing 215 employed patients with GO. Among those, 12 (6%) and 47 (22%) were considered permanently or temporarily disabled, respectively. Patients with optic neuropathy were almost twice as likely to be disabled at work than patients without optic nerve compression. The overall frequency of work disability was 20.9% in patients without diplopia, while greater percentages, i.e., 41%, 42% and 74% were found in those affected by intermittent, inconstant, and constant diplopia, respectively [36].

The growing number of survivors of thyroid cancer raised increased attention towards the late effects of disease and treatment. Despite the long-term survival rate, patients more often suffered from neurophysiological short-comings like fatigue [44], anxiety, and psychological distress [45] compared to the unaffected population. In this regard, a Swiss study comparing DTC patients and their partners, found that, although most cancer survivors continued to work, the majority reported some level of work impairment within the first year post diagnosis, with about 24% of patients failing to handle the same workload as prior to their diagnosis [34]. Significant differences were found between patients with a reduced workload and those without changes in terms of higher levels of fatigue and reduced physical quality of life [12,44]. Female patients need more time to return to work after hospital discharge and a significantly greater percentage reported a reduced workload compared to males. Thyroid cancer survivors in the United States experienced work difficulties as well as poor productivity [33]. This issue was recently assessed in a study performed on 1743 patients with a history of thyroid cancer. Among the investigated patients, 59.6% lost productivity at work. Employment status was also associated with health-related quality of life issues. Individuals reporting lost productivity at work due to cancer diagnosis claimed worse fatigue and social functioning [33]. This may be also related to the inability to change jobs or get a new job due to thyroid cancer diagnosis. The above-mentioned results are in line with those reported in a previous study carried out in the US that demonstrated that among 381 thyroid cancer survivors, 63% of respondents were working and 7% indicated that they were unable to work. In this latter group, a significantly longer interval from diagnosis was assessed (16.2 ± 15.2 vs. 8.1 ± 9.7 years) [27]. No association between direct therapeutic exposures to cancer and interference with the physical or mental tasks required for their work among survivors of thyroid cancer has been identified [32].

A German study investigated the effects of hypothyroidism secondary to thyroid hormone withdrawal in 130 patients with DTC on work ability. Among those, 49 of 59 patients (83%) working away from home reported impaired ability to work during the withdrawal period. Significant, moderate and mild implications on work ability were reported in 44%, 27%, and 16% of the working subgroup, respectively, demonstrating that morbidity derived from the secondary hypothyroidism can affect productivity and work ability [30]. Schroeder et al. [40] showed that 74% of patients had difficulties in performing work after withdrawal, when compared with 18% of those on LT4 and 21% of those undergoing rhTSH stimulation. Among employed patients or trainees with thyroid cancer, 9.1% were able to exercise their job with restrictions in the last four weeks before their second control visit at 9–18 months post-thyroidectomy [25]. An exploratory Canadian study on fatigue and physical activity in thyroid cancer survivors showed that a substantial percentage of those patients, about 4 out of 10, claimed moderate or severe fatigue. Disabled people reported significantly more interference with normal work due to fatigue than other people [35]. These results complement those of Tamminga et al. [29] who reported that a higher level of fatigue was an independent predictor of unemployment in long-term survivors of thyroid cancer. Furthermore, this study demonstrated that 74 of 223 thyroid cancer survivors have been forced to change their employment status due to cancer, particularly as concerns working a lower number of hours

### 3.4. Labour Market Income and Disability Pension

As thyroid diseases can affect ability to work, it may be argued that thyroid patients may have a higher risk of labour market income changes and disability pension as addressed in several studies [6,10,31,33,46] (Table 4). Brandt et al. [46] demonstrated that hyperthyroid patients had an 88% increase in risk of receiving a disability pension compared to controls. When the cause of the disease was analyzed with respect to the possibility to receive a disability pension, a significantly increased risk was evident only in toxic nodular goiter, but not in Graves’ disease patients. Hyperthyroid patients also had a significantly lower mean income before, as well as after, the diagnosis of the disease. A lower progress in income at the same time points was much higher in Graves’ disease than in TNG patients. Analyses of twin pairs discordant for hyperthyroidism demonstrated an increased risk of disability pension and a significant difference in income increase favoring euthyroid twin, thus supporting a causal relationship with hyperthyroidism. These results are in line with those obtained in a smaller, previous investigation that demonstrated a significantly higher risk of disability pension in hyperthyroid patients within the first year from the diagnosis [10]. During subsequent years, thyroid patients differed from controls, primarily due to a higher risk of disability pension among patients with GO. Concerning the possible implications of GO on earning capacity, among 192 patients in gainful employment, the earning capacity of 124 (64.5%) was not impaired at any stage, while 41 (21.3%) and 12 (6.2%) suffered temporary and permanent impairments, respectively. Impaired earning capacity was associated with the severity of GO and the manifestation of diplopia, while no significant correlation was found for proptosis [18].

Concerning hypothyroidism, another observational cohort study could demonstrate that having this diagnosis before the age of 60 years was associated with loss of labour market income and with an 89% increased risk of receiving a disability pension [6]. A significantly higher fraction of cases than controls received a disability pension at an age that was significantly lower for patients (48.8 years) compared to controls (53.1 years). The risk of receiving disability pension was not affected by pre-existing comorbidity, supporting the role of hypothyroidism per se in causing disability pension. As regards labour market income, cases received significantly lower income before and after the diagnosis of hypothyroidism compared to controls, and experienced also a lower increase between these 2 time points. When the employment status of thyroid cancer survivors was assessed, Mongelli et al. [33] reported that 18.5% of the 1753 investigated subjects was retired, 42.5% lost job income, while 6.6% reported to receive disability benefits. This latter condition was associated with worse pain interference. Additionally, financial difficulties were reported by 43% thyroid cancer survivors and were associated with higher anxiety, and depression. Ratzon et al. [31] showed that a significantly greater percentage of cancer patients claimed a reduced income compared to controls at 2 (49.0% vs. 38.4%) and 4 (47.7%% vs. 37.1%) years after the diagnosis. Slaweta et al. [42] found that among patients who had undergone thyroid surgery for cancers, among those professionally active who had one or two post-surgery complications, 15% received an invalidity pension with a mean age at the time of retirement of 48.5 years.

## 4. Discussion

This review represents the first attempt to provide an overview on the possible implications of thyroid diseases on patients’ work functioning. To gain deep insight into the “working status” of thyroid patients is absolutely important for a correct and comprehensive disease management. To understand which pathological features may affect the employment and work ability of patients may support the adoption of suitable management strategies able to prevent negative consequences on their occupational lives. This may overall improve social inclusion of patients [22], self-esteem [47], financial situation [24], therefore, supporting a better quality of life [21]. Although the limited and fragmented data available do not allow to extrapolate definite conclusions, interesting issues emerged from our analysis that may provide guidance for future investigations.

Concerning the risk of unemployment in patients with functional thyroid diseases, the lack of adequate investigations prevents one to define suitable relationships. Only one study, in fact, found no significant differences in patients affected by nontoxic goiter, hyperthyroidism, GO and autoimmune hypothyroidism, in the first year after diagnosis, compared to unaffected controls [10]. Moreover, conflicting percentages of employment were reported in GO and hyperthyroidism treated patients [11,18,19]. Specifically-focused, prospective studies in this regard should be performed to clarify this aspect also considering longer periods of investigation and possible changes in disease phenotypes over time. When thyroid cancer survivors were analyzed, about one third of patients resulted in unemployed status [29,31]. This condition was related to a higher age at diagnosis and a lower educational level. Additionally, also an experienced higher level of fatigue has been reported as a possible predictive factor for unemployment [29,35]. However, the causal relationship in such association remain to be understood. In fact, it may be argued that cancer survivors with fatigue may be at risk of loss of employment due to this symptom, or may suffer from other comorbidities resulting in fatigue and inability to work, or that the lack of employment may in part, contribute to fatigue, loss of life structure, worsening emotional and financial distress or other factors [29].

Sick leaves have been also investigated as a possible indicator of morbidity. The findings that hyperthyroid patients had a higher risk of long-term sick leave compared to controls [10], and the reported increasing number of days-lost positively related to the severity of GO [36], support the idea that the numbers of days patients stayed at home well resemble the severity of the disease, and its impact on patient health. Concerning the possible function of sick leaves as “occupational indicators” in monitoring adequate treatment strategies for a comprehensive and inclusive management of thyroid patients, no conclusive data are currently available on hyperthyroidism. The significant sick leave reduction experienced by GO eu-hypothyroid patients compared to hyperthyroid ones may suggest that a better control of the pathology may be associated with a lower number of days lost [11]. However, when different treatment options, i.e., antithyroid drugs, surgery or radioiodine therapies, were investigated in relation to their impact on absences from work, no significant differences could be detected [37]. In this perspective, it cannot be excluded that, although hyperthyroidism remission was achieved, some illness complaints, i.e., vegetative, cognitive neuro-psychological and emotional disturbances, may persist, thus preventing patients coming to work. Conversely, sick leaves was reported as a possible indicator of therapeutic effectiveness, in the case of radiation exposure for differentiated thyroid cancers [32], when the employment of the rhTSH therapy in the current practice of the RTI was assessed [41], as well as when such stimulation procedure was adopted to avoid thyroid hormone withdrawal in the diagnostic follow-up of cancer patients treated with thyroidectomy [25,28,30].

As regards work ability, although the majority of hyperthyroid patients were reported to be able to effectively manage their jobs, more than one third of the affected patients experienced difficulties in maintaining their work [19] or could not work at all for some periods of time [43]. In some cases, patients have been officially registered as completely or partially disabled [18,19]. However, differences in investigation tools employed to assess this peculiar aspect of the professional life, prevent to obtain comparable data. In this perspective, validated and more homogenous tools should be employed to assess patient perception of their ability to work and impairments. Importantly, impairment in work ability was reported also when hyperthyroidism remission was achieved requiring additional research to define which pathological factors can be associated with these adverse occupational outcomes. Concerning gender related differences in work ability, only Neudeck et al. [34] pointed out differences in return to work time and work-related impairment between male and female patients treated for thyroid cancers, while Ratzon et al. [31] reported an increased risk for unemployment only in female cancer survivors. To extrapolate specific gender related data is a quite challenging issue, as most of the studies fail to assess such interesting aspect. This may be due to the greater prevalence of thyroid disorders in females compared to male subjects. The female to male ratio, in fact, ranges from 1.5:1 for thyroiditis, to 5:1 for toxic nodular diseases and 8:1 for Graves’ disease, up to 10:1 for hypothyroidism [48]. In line with these epidemiology data, the percentage of female patients enrolled in the reviewed studies is generally greater than 70%, therefore limiting a suitable comparison between genders which requires deeper attention. 

In patients affected by GO, disease severity and diplopia resulted significantly associated with occupational disability and earning capacity, while no association was detected for proptosis [18]. This indicates that functional impairments played a greater role than cosmetic ones in work ability. Severely affected patients, apart from occupational disabilities, suffered from contemporary impairments into the everyday functioning and/or into their own self-perception as a result of GO, and needed more psychotherapy compared to less severe cases. This supports the need for an early prevention of disease complications and the adoption of an optimal interdisciplinary care for GO patients. Endocrinologists, ophthalmologists, specialists in nuclear medicine, radiologists, and occupational physicians should work in close collaboration addressing the different aspects of the disease that may impact personal and professional life of patients. It is also important to note that the potential problems seem to be most pronounced within the first year of diagnosis and diminished with time, presumably because of treatment effects and/or effective coping by patients [34].

In regards to DTC, although the excellent prognosis, the majority of patients reported some level of work impairment within the first-year post diagnosis [27,33,34]. However, no data are available concerning the impact that thyroid cancers may have on work functioning with respect to the disease characteristics, such as the histological type, the applied treatment, as well as age at and time from the diagnosis. All these aspects, in fact, may play an influence on occupational outcomes. A higher level of fatigue and reduced physical quality of life were associated with DTC related impairments [33,34]. It should be considered, also, that inability to change jobs or get a new job due to thyroid cancer diagnosis was associated with worse fatigue, pain interference, and social functioning [33]. This suggests that DTC patients should be screened and, when necessary, treated for fatigue. Evidence-based nonpharmacological treatment options may be considered, such as psychoeducational interventions, physical activity/exercise, mindfulness based stress reduction, and cognitive-behavioral stress management together with pharmacological possibilities.

Work disability may impact work productivity and work role function among patients who, although remaining on the labour market, are not firstly involved in promotion or wage increase [6,31,33,46]. Individuals diagnosed with thyroid diseases, i.e., hyper- and hypothyroidism, had an increased risk of receiving disability pension compared to controls [10,18,46], and also patients remaining on the labour market had a lower progression in income compared to controls [6,31,33,42]. These effects persisted after controlling for co-morbidity, level of education, and twin pairs discordant for hyperthyroidism indicating a causal link between work disability and disease diagnosis [46]. Thyroid phenotype had substantial effect on the results from income analysis. Overall, hyperthyroidism was associated with a lower increase in income compared to the controls, but it was much higher in Graves’ disease than in TNG. The more acute and severe toxic state in Graves’ disease and/or the accompanying ophthalmopathy, together with the fact that this disease primarily affects younger individuals at higher risk of missing work training or receiving a promotion, may explained such difference. In regards to a possible relationship between disability pension and work ability or sick leave, some preliminary results may suggest such an association. Slaweta et al. [42] found that cancer patients who received disability pension were those with two or more complications after thyroid surgery who showed, at the same time, a longer average period of absence from work. Comparably, Nexo et al. [10] reported that, within the first year after hyperthyroidism diagnosis, patients experienced the greatest risk for long term-sickness absence as well as the highest risk for disability pension. Cancer survivors with some impairment at work, reported worsen fatigue, pain interference and social functioning. Such factors were also predictive for lost productivity, and disability or social security benefits [33]. However, a quantitative analysis of the relationship cannot be performed, considering also that most of the revised papers failed to investigate different occupational outcomes to develop a suitable association.

Importantly, all the articles addressing unemployment or work disability in thyroid patients failed to include work-related factors such as type of occupation, risk factors experienced, i.e., chemical, physical, and biological risks, physical workload as well as job organizational issues, including work-related stress or shift works, in their analysis. For that reason, the obtained results might not be optimal in explaining whether, and under which mode of action, some of these factors may have an impact on thyroid patients therefore specifically leading to unemployment or work impairment. From an occupational health perspective, this may limit an adequate risk assessment in the workplace and the possibility to adopt preventive and protective measures to achieve work conditions suitable also for thyroid patients.

Finally, some limitations emerged from our revision should be pointed out to plan future methodologically adequate investigations able to provide more informative data. The cross-sectional design of the majority of the reviewed studies do not allow to make causal inferences in the relationship between the disease and occupational impairments. In this regard, prospective investigations assessing diverse thyroid disorders and different work-related outcomes over time should be strongly pursued. Currently available information does not allow to extrapolate conclusions concerning the work functioning impact of different hypo-hyperthyroid functional disorders. Future studies are necessary to make suitable comparisons between such diverse conditions, and also in relation to the age and gender related features of affected patients. In terms of providing prognostic information to patients, it is important to be able to differentiate between impact on work life during the first year post-diagnosis, when treatment is initiated, and on work life in subsequent years, also in relation to possible clinical changes in thyroid disease, comorbidity, treatment effectiveness and personal coping strategies developed over time. Finally, future studies should provide a multistate analysis to simultaneously investigate changes affecting several work-related outcomes, i.e., employment status, sickness absence, work ability and disability pension, representing competing outcomes. Selection bias may have occurred in the reviewed studies because the cases enrolled in specialized centers may be more severe thus experiencing more negative professional consequences. Therefore, future studies should extend the survey to patients with diverse severity of disease as thyroid pathologies generally manifest as a graded phenomenon ranging from subclinical to clinically evident alterations. This may be helpful in extrapolating conclusions that would be of general validity both for physicians who work in endocrine specialized areas, but also for primary care and occupational physicians who may care patients with all the spectrum of disease manifestations. In this perspective, future studies should investigate occupational outcomes also in relation to a clinical or biochemical classification of patients, as well as in relation to the given therapy and rate of relapses. Concerning the possibility that residual complaints may affect patients also in conditions of thyroid functional remission, specific attention should be focused to understand the relationship between actual duration of functional thyroid disorders and the duration of such impairments in order to define the impact on working life and suitable management strategies.

## 5. Conclusions

Although preliminary, the described results stress the importance for clinicians to carefully assess the work function of their patients as part of their clinical management. To this aim, a deeper investigation seems necessary to understand the implications that different thyroid disorders, with different severity manifestations may have on work-related outcomes, in terms of i.e., employment, days lost from work, disability in performing job tasks, as well as earning capacity. Such changes should be assessed in prospective studies to have an idea of their trend over time in regard to disease development and clinical course. Occupational related outcomes should be also put in relation with risk factors experienced in the workplace. To understand which job task features may be more dangerous for such hyper-susceptible individuals, may support a tailored risk assessment process and suitable management strategies. All these efforts will guarantee thyroid patients a comprehensive, interdisciplinary management that may improve disease outcomes, as well as their personal, social and professional life. Additionally, from a public and occupational health perspective, society may benefit from such approach through a reduction of direct and indirect costs caused by these widespread endocrine pathologies.

## Figures and Tables

**Table 3:** Studies addressing the impact of thyroid disease on work ability.

Study Location (Analysed Period)	Population Investigated (Number) and Age	Additional Information	Results	Quality Rating According to NOS	References
Stockholm, Uppsala, Sweden (1983–1990)	See Torring et al. [37]	Clinical data YA received anti-thyroid drugs for 18 months (medical treatment) or subtotal thyroidectomy (surgical treatment). OA received medical, surgical, or radioiodine treatment. Follow-up time: at least 48 months.	Work ability: During the last month prior to treatment 65% reported that they could manage their professional work. Treatment did not affect at all or very little work ability in 63%; 19% could not work at all or very little for 1 month (64%), 2 to 3 months (22%) or more. Eye problems were responsible for serious limitations in 20% and of minor limitations in 42% of patients complaining such manifestation.	Satisfactory	Ljunggren et al. [43]
Amsterdam, Netherlands (1994–2000)	Patients treated for hyperthyroidism (n. 303; F: 87%; mean age: 44.0 ± 11.2 years).	Clinical data Hyperthyroidism treatment: surgery (n. 31; 10%); 131I (n. 151; 50%); thyreo-static drugs (n. 78; 26%); levothyroxine (n. 166; 55%). A total of 119 patients (40%) reported no drug treatment at the survey time.	Work ability among full-time employees (n. 107): unable to resume the same work even after remission of hyperthyroidism (35.3%); completely or partially disabled (29.5%).	Satisfactory	Fahrenfort et al. [19]
Copenaghen, Denmark (2007–2013)	See Table 1	See Table 1	Patients affected by Graves’ disease, autoimmune hypothyroidism and other thyroid diseases had significantly lower scores compared to the general population on the global work ability item (G_WAI). Patients rated their work ability worse within the first year of diagnosis.	Satisfactory	Nexo et al. [10]
Mainz, Germany (2006–2008)	See Table 1	See Table 1	Work ability: unfit to work n. 192; never unfit to work/disabled (n. 124; 64.5%); unfit to work for a maximum of 1 month (n. 38; 19.7%), 2–3 months (n. 10; 5.2%), 4–6 months (n. 6; 3.1%), 7–9 (n. 1; 0.5%) and 10–12 months (n. 1; 0.5%), permanently disabled (n. 12; 6.2%). Earning capacity: (n. 192 EO patients) not impaired at any stage (n. 124; 64.5%); temporary impairment (n. 41; 21.3%); permanently impaired (n. 12; 6.2%).	Satisfactory	Ponto et al. [18]
Mainz, Hannover, Germany (2005–2009)	See Table 2	See Table 2	Work ability (among 215 employed GO patients): temporarily disabled n. 47 (22%); permanently disabled n. 12 (6%); lost job n. 5 (2%); early retired early n. 9 (4%). Clinical factors associated with impaired work ability: optic neuropathy and diplopia (significant association).	Satisfactory	Ponto et al. [36]
Olten, Zurich, Luzern, Switzerland (2009–2016)	See Table 1	See Table 1	47 of 66 patients (71.2%) and 22 of 38 partners (57.9%) felt impaired with respect to their work ability during the first year after the diagnosis of DTC. Patients demonstrated significantly less workload achievable (24.2% vs. 2.6%) and getting tired faster (60.6% vs. 7.9%) compared to controls.	Satisfactory	Neudeck et al. [34]
Chicago, United States	See Table 1	See Table 1	N. 1033 (59.6%) reported to have lost productivity at work. Clinical factors associated with impaired work ability: worse fatigue and social functioning. Labour market income: lost income at work n. 737 (42.5%); receive disability benefits n. 115 (6.6%).	Satisfactory	Mongelli et al. [33]
Houston, Texas	See Table 1	See Table 1	N. 37 (7%) of 324 cancer survivors were unable to work. Individuals unable to work had a significantly longer interval from diagnosis than other unimpaired workers (16.2 ± 15.2 vs. 8.1 ± 9.7 years, respectively).	Good	Schultz et al. [27]
United States (2019)	See Table 1	See Table 1	Among patients employed at some time between cancer diagnosis and study participation, no association was found between direct therapeutic exposures to cancer and interference with the physical or mental tasks required for work.	Good	Ketterl et al. [32]
Würzburg, Cologne, Germany (1992–2001)	See Table 1	See Table 1	Among employed patients n. 49 (83%) reported some impairment; n. 26 (44%) considerable, n. 16 (27%) moderate impairment, n. 7 (16%) mild impairment.	Satisfactory	Luster et al. [30]
United States	Patients with thyroid carcinoma (n. 229; F: n. 148; mean age: 47 ± 16)	Clinical data The patients were studied at three time points: at baseline on levothyroxine suppressive therapy, after rhTSH while on levothiroxine suppressive on the day of the WBS, or after withdrawal of thyroid hormone on the day of the WBS.	74% of patients had difficulty performing a job because of physical health after withdrawal, compared with 18% or 21% in baseline levothyroxine suppressive therapy or after rhTSH.	Unsatisfactory	Schroeder et al. [40]
Wuerzburg, Germany (2008)	See Table 1	See Table 1	9.1% of patients was able to exercise their job with restrictions in the last four weeks before control 2.	Satisfactory	Dueren et al. [25]
Toronto, Canada (2015)	See Table 1	See Table 1	Clinical factors significantly associated with impaired work ability: worsening fatigue.	Satisfactory	Alhashemi et al. [35]
Amsterdam, Gronigren, Nijmegen, Eindhoven, The Netherlands (1990–2008)	See Table 1	See Table 1	One third (33%) of n. 223 thyroid cancer survivors reported work changes due to the disease: working less hours (16.6%); being disabled (9%); being fired (5%); stopped working (4%); re-educated (3%), early pension (1%).	Good	Tamminga et al. [29]

EO, endocrine orbitopathy; DTC, differentiated thyroid cancer; GO, Graves’ orbitopathy; OA, old adults; RhTSH, recombinant human thyroid stimulating hormone; TAO, thyroid-associated orbitopathy; WBS, whole body scan; YA, young adults.

**Table 4:** Summary of the studies addressing the impact of thyroid disease on labor market income and disability pension.

Study Location (Analysed Period)	Population Investigated (Number) and Age	Additional Information	Results	Quality Rating According to NOS	References
Kielce, Poland (2002–2007)	See Table 2	See Table 2	Disability pension: 4 subjects (15% of professionally active subjects) with one or two coexisting complications (average age: 48.5 years). Three subjects (11%) retired early (average age: 59 years).	Unsatisfactory	Slaweta et al. [42]
Copenhagen, Denmark (1994–2011)	See Table 1	See Table 1	Significantly increased risk for hyperthyroid patients (<1 year HR 4.15; 95%CI 1.76–9.77) and GO patients (>1 year HR 4.40; 95%CI 2.61–7.42).	Very good	Nexo et al. [10]
Odense, Denmark (1977–2006)	Hypothyroid cases (n. 1745; mean age: 45.4 years). Controls (n. 6980; mean age: 45.4 years)	Clinical data Subjects with hypothyroidism had significantly higher comorbidity than controls. No differences in educational level.	Cases showed lower income before and after diagnosis compared to controls. Significantly lower increase in income from 2 years before and 2 years after the diagnosis (1605 € lower). A significantly 89% higher risk of receiving disability pension compared to controls and mean age at disability pension was significantly lower in cases.	Very good	Thvilum et al. [6]
Odense, Denmark (1978–2006)	Hyperthyroid patients (n. 1942; F: 83%; mean age at diagnosis: 44.5 years; mean age at disability pension: 47.4). Controls (n. 7768; F: 83%; mean age at diagnosis: 44.5 years; mean age at disability pension: 47.9 years)	Clinical data Graves’ disease (n. 442); nodular toxic goiter (n. 195) *Occupational data* Disability pension was defined as retirement before the age of 60.	From 2 years before to 2 years after the diagnosis was significantly lower (1189 €) in affected patients compared to controls. This difference was more pronounced in GD (2539 €) compared to TNG (132 €). Significantly 88% increased risk of receiving disability pension in cases compared to controls.	Very good	Brandt et al. [46]
Israel (1998–2011)	See Table 1	See Table 1	Significant difference in the frequency of decreased labour income between cases and controls 2 years (49.0% vs. 38.4%, respectively) and 4 years after diagnosis (47.7% vs. 37.1%, respectively).	Very good	Ratzon et al. [31]
Chicago, United States	See Table 1	See Table 1	Labour market income: lost income at work n. 737 (42.5%); receive disability benefits n. 115 (6.6%).	Satisfactory	Mongelli et al. [33]
Mainz, Germany (2006–2008)	See Table 1	See Table 1	Among 192 patients in gainful employment, the earning capacity of 124 (64.5%) was not impaired at any stage, while 41 (21.3%) and 12 (6.2%) suffered temporary and permanent impairments, respectively.	Satisfactory	Ponto et al. [18]

GD, Graves’ disease; GO, Graves’ orbitopathy; HR, hazard ratio; TNG, toxic nodular goiter.
